# Effects of Short-Term Broccoli Powder Supplementation on Acute Oxidative Stress and Recovery Following a Metabolically Demanding Exercise Session

**DOI:** 10.3390/antiox15030379

**Published:** 2026-03-18

**Authors:** Leonardo Cesanelli, Tomas Venckunas, Petras Minderis, Viktorija Maconyte, Arvydas Stasiulis, Audrius Snieckus, Mantas Mickevicius, Dalia Mickeviciene, Sigitas Kamandulis

**Affiliations:** 1 Institute of Sport Science and Innovations, Lithuanian Sports University, 44221 Kaunas, Lithuania; petras.minderis@lsu.lt (P.M.); viktorija.maconyte@lsu.lt (V.M.); audrius.snieckus@lsu.lt (A.S.); mantas.mickevicius@lsu.lt (M.M.); dalia.mickeviciene@lsu.lt (D.M.); sigitas.kamandulis@lsu.lt (S.K.); 2 Department of Health Promotion and Rehabilitation, Lithuanian Sports University, 44221 Kaunas, Lithuania; arvydas.stasiulis@lsu.lt

**Keywords:** sulforaphane, bioavailability, lactate, recovery, VO_2_max

## Abstract

**Background/Objectives**: This study aimed to evaluate the effects of short-term broccoli powder supplementation on metabolically demanding exercise performance, muscle power, and blood lactate recovery. It also investigated broccoli powder-derived sulforaphane bioavailability and its effects in attenuating exercise-induced oxidative stress. **Methods**: Seventeen healthy males (age 23.8 ± 4.9 years, height 182.3 ± 6.1 cm, weight 80.0 ± 12.8 kg), in a double-blind crossover design, three weeks apart, consumed ten standard doses of either broccoli powder or spinach powder as a placebo over a period of 2 weeks. They then performed a maximal progressive cycling task with concomitant analysis of expired gas composition. Plasma malondialdehyde (MDA) level was measured before and 60 min after the completion of the task, and blood lactate and muscle power (counter-movement vertical jump (CMJ) performance) were measured before and up to 60 min after exercise. **Results**: The main findings were that despite urine sulforaphane output being markedly higher following broccoli supplementation (*p* < 0.05), which confirms effective absorption and systemic availability of the compound, this did not influence exercise-induced changes in plasma MDA concentration, blood lactate dynamics, exercise test performance, or functional recovery measured as muscle power via CMJ performance (*p* > 0.05). **Conclusions**: In conclusion, broccoli powder supplementation, despite efficient delivery of sulforaphane, does not seem to either acutely affect performance or modify oxidative stress and recovery from metabolically demanding exercise.

## 1. Introduction

Intense exercise transiently increases oxygen consumption and reactive oxygen species (ROS) production through mitochondrial respiration and other metabolic pathways [1,2]. Although low to moderate levels of ROS act as important signaling molecules for physiological adaptation, excessive accumulation, especially over prolonged periods, can lead to oxidative damage to lipids, proteins, and nucleic acids [3,4]. The balance between ROS generation and antioxidant defense systems—both enzymatic (e.g., superoxide dismutase, catalase, glutathione peroxidase) and non-enzymatic (e.g., vitamins, polyphenols)—is therefore critical for maintaining redox homeostasis during and after exercise [4,5,6,7].

Given the involvement of ROS in exercise-induced fatigue and recovery [1,2,3,8,9,10], antioxidant supplementation has been widely explored as a strategy to mitigate oxidative stress and improve performance [4,11,12,13,14]. However, while some studies indeed report reductions in oxidative biomarkers and improved recovery following antioxidant intake, others show no or even adverse effects of exogenous antioxidant supplementation on long-term training adaptation due to blunting of exercise-induced adaptive signaling [11,12,15,16]. Recent research has thus shifted toward modulatory approaches using natural compounds capable of supporting endogenous antioxidant capacity rather than directly scavenging free radicals [17,18,19,20].

Cruciferous vegetables such as broccoli are rich in glucosinolates, among which glucoraphanin is the precursor of sulforaphane (SFN), a potent isothiocyanate with well-documented cytoprotective properties [21,22,23]. Upon enzymatic hydrolysis by myrosinase or intestinal microbiota, glucoraphanin is converted to SFN, which, when transported into the cells, activates the nuclear factor erythroid 2-related factor 2 (Nrf2) signaling pathway [24,25,26]. This pathway upregulates the expression of phase II detoxifying and antioxidant enzymes, including heme oxygenase-1 (HO-1), NAD(P)H quinone oxidoreductase-1 (NQO1), and glutathione synthesis enzymes [24,26,27,28], and may enhance the body’s intrinsic defense against oxidative and inflammatory stress by sustained antioxidant effect rather than an acute radical scavenging [24,25,26]. While this transcriptional pathway represents the best-characterized mechanism of sulforaphane action, the present proof-of-concept study was not designed to examine solely Nrf2-dependent adaptations; rather, it evaluated whether short-term supplementation, via the cumulative effects of repeated broccoli supplementation, could influence physiological responses to an acute, metabolically demanding exercise bout.

In addition to its antioxidant and cytoprotective functions, broccoli supplementation may also exert prebiotic effects by modulating the gut microbiome composition. Increased microbial diversity and the enrichment of taxa associated with improved nutrient metabolism and intestinal barrier integrity have been reported following higher intake of cruciferous vegetables [29,30,31]. Such adaptations could facilitate more efficient gut–blood exchange of metabolites, including lactate and free fatty acids, thereby potentially enhancing systemic metabolic responses, exercise capacity, and post-exercise recovery [32].

While the health-promoting properties of sulforaphane are well recognized, its effects in the context of exercise-induced oxidative stress remain insufficiently characterized, especially in a healthy, normally active population [13,20,33,34,35]. Previous studies investigating antioxidant supplementation during physical exercise have produced mixed outcomes, often depending on the intensity and duration of the exercise stimulus as well as the dosage and timing of the supplement [4]. Notably, most experimental models have used strenuous exercise protocols designed to elicit pronounced oxidative responses, which may not represent typical conditions for recreationally active individuals [3,4,36]. Consequently, evidence regarding the efficacy of sulforaphane supplementation under ecologically valid, metabolically demanding exercise conditions remains limited. The current study was therefore designed to prioritize ecological validity, which might have subsequently limited the ability to detect subtle biochemical modulations, even if biologically present.

The present study aimed to evaluate the effects of short-term broccoli powder supplementation on metabolically demanding exercise performance as well as muscle power and blood lactate recovery dynamics in healthy adults using a double-blind crossover design. We also investigated the sulforaphane bioavailability of the supplementation, and its effects to attenuate post-exercise oxidative stress measured as changes in plasma malondialdehyde (MDA) concentration. Previous studies showed that sulforaphane supplementation for 14 days was shown to be a sufficient period to effectively blunt muscle damage in response to eccentric exercise [33], and even as short as 7 days of diet supplementation with mustard seed powder-enriched broccoli sprout juice during a concomitant highly metabolically demanding repeated sprint exercise training period was effective in decreasing oxidative and metabolic stress [20]. We therefore hypothesized that consumption of broccoli powder enriched with myrosinase would not only render high sulforaphane bioavailability, which would be evident by increased sulforaphane excretion via urine, but a short-term (2 weeks) supplementation period would be sufficient to attenuate oxidative stress responses to an acute bout of metabolically demanding exercise without impairing physiological responses, performance, and recovery. Longer-term supplementation effects were outside the scope of this study; however, such an investigation would be of interest because of the potential additional sulforaphane effects on consumers’ lifestyles, which may affect exercise capacity, acute exercise-induced physiological perturbations, and subsequent recovery.

## 2. Materials and Methods

### 2.1. Participants

Twenty healthy males aged 18–37 years were recruited. They had no illnesses or injuries in the month preceding data collection and were engaged in organized physical activity no more than twice per week. Three participants dropped out due to acute infectious disease or changed life circumstances, leaving 17 participants for the analyses (mean ± SD: age 23.8 ± 4.9 years, height 182.3 ± 6.1 cm, weight 80.0 ± 12.8 kg). Because no effect size estimates from comparable crossover trials were available, the sample size was determined pragmatically based on feasibility, targeting at least ~15 completers. A within-subject crossover design was employed to reduce inter-individual variability in oxidative stress and performance responses to exercise. A post hoc sensitivity analysis (α = 0.05, 1 − β = 0.80) indicated that the final sample size (*n* = 17) provided sufficient power to detect medium-sized Time × Intervention interaction effects (partial η^2^ ≥ 0.06). Participants maintained their regular diets and daily routine. Each participant read and signed a written informed consent form. The study was conducted in accordance with the Declaration of Helsinki, approved by the Lithuanian Sports University Ethics Committee (protocol code BI-TRS (M)-2024-703, date of approval: 30 October 2024), and registered retrospectively at ClinicalTrials.gov (3 March 2026; ClinicalTrials.gov; ID: NCT07454265).

### 2.2. Study Design and Measurements

This study was a double-blind, crossover design, with neither the participants nor the investigators directly in contact with the participants aware of the intervention condition. The input order was randomized, and a 3-week washout period was applied between conditions. Participants were asked to complete questionnaires regarding their consumption of stimulating drinks (coffee, tea, energy drinks, alcohol), broccoli-related foods (Brussels sprouts, mustard greens, leafy cabbage, turnips, cabbage, cauliflower, horseradish, arugula, radishes), and supplements (proteins, BCAA, omega-3, carbohydrates, creatine) within the 24 h preceding each exercise test.

Intervention arm: The intervention consisted of a single 35-mL scoop of broccoli powder (BrocAffex; 99.5% broccoli powder and 0.5% mustard seed powder), corresponding to 10 g of supplement and delivering 320 μg of glucoraphanin per serving. The supplement was mixed with 300 mL of chocolate oat milk (13 participants) or 300 mL of orange juice (4 participants who did not like chocolate oat milk and broccoli cocktail).

Placebo arm: 1/2 × 35 mL scoop of dried spinach powder blended with 300 mL of chocolate oat milk (13 participants) or orange juice (4 participants).

Dosing schedule (10 doses in total): 14, 12, 10, 8, 6, 4, 3, 2, and 1 day, and 3 h before the exercise challenge.

**Figure 1:**
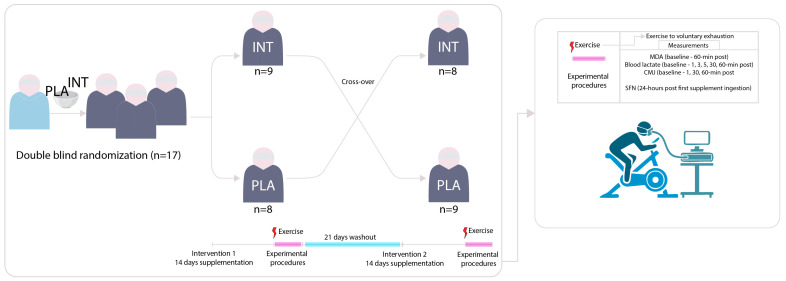
Schematic representation of the study design. Subjects were randomly and blindly assigned to either the supplementation intervention (INT) or the placebo group (PLA). The supplementation period commenced 14 days prior to the exercise protocol and concluded 3 h before testing. The exercise protocol consisted of an incremental bike ergometer test to voluntary exhaustion. Urine samples for the analysis of sulforaphane (SFN) were collected 24 h after the ingestion of the first supplement dose. Measures of malondialdehyde (MDA) concentration were taken before and after the exercise test. Blood lactate and countermovement jump (CMJ) height were assessed at multiple time points following the exercise protocol.

A fresh beverage was made as a cocktail using a shaker just before each consumption, was drunk within several minutes, and a glass of water (300–500 mL) was taken within 30 min after consumption. A representation of the study design is reported in Figure 1.

### 2.3. Urine Collection and Analysis

All urine produced by the participant was collected over a 24 h period after ingestion of the first supplementation dose. The urine was collected into a clean plastic container and kept in a cool place, then delivered to the lab within 2 h at the end of a 24 h period of collection. The urine volume was measured, and aliquots were taken and kept at −80 °C for subsequent sulforaphane (SFN) analysis by LC-MS (liquid chromatograph Shimadzu LC-30AD, mass spectrometer LCMS-2020, Shimadzu Corporation, Nakagyo-ku, Kyoto, Japan). Urine was analysed in all twenty participants from the Broccoli supplementation condition and in five randomly selected participants from the Placebo condition.

### 2.4. Exercise Tests

*Metabolically demanding exercise task.* Incremental ramp cycling exercise up to voluntary exhaustion was implemented using the following protocol: 4 min at 40 W, after which power was continuously increasing by 1 W/3 s ramp (i.e., by 20 W each minute). A portable breath-by-breath analyser (Cortex Metamax 3B, Cortex Biophysik GmbH, Leipzig, Germany) was used, and heart rate (HR) was monitored (Polar H10, Kempele, Finland) throughout the test. Participants were pedalling at ~70 rpm and were required to cycle until exhaustion with standardized verbal encouragement during the last stages of the test. The following criteria were used to verify that the maximal oxygen uptake (VO_2_max) was attained: peak HR reached at least 90% of the age-predicted maximal HR (220 minus individual ages in years), and the respiratory exchange ratio reached > 1.1. The following peak parameters were measured: HR, oxygen consumption (VO_2_), pulmonary ventilation (VE), breathing frequency, and respiratory exchange ratio (RER, CO_2_/VO_2_). VO_2_max and HRmax were determined as the highest values of 20-s rolling average during the latest stages of the test. The test failure criterion was the inability to maintain cadence above 60 rpm for longer than 5 s despite verbal encouragement.

*Muscle power test.* Countermovement jump (CMJ) height was measured before (baseline), and then 1 min, 30 min, and 60 min after the metabolically demanding exercise task. Before the first (baseline) and the last (60 min after the metabolically demanding exercise task) muscle power testing time point, a short, standardized warm-up consisting of 5 min of very light cycling at 50 W on the cycle ergometer (Ergo Line, Medical Measurement Systems, Bitz, Germany) and 2 min of low-intensity dynamic stretching was conducted. Each jump began from an upright standing position, followed by squatting to approximately 90° knee angle before immediately jumping vertically off the ground as high as possible. A 20-s interval was maintained between each of the attempts, and participants kept their hands on their waist during the jumps. The CMJs were performed using a photoelectric cell system (Optojump, Microgate, Italy), and the best result from three to four attempts was taken for analysis.

### 2.5. Blood Analyses

Capillary blood lactate was measured from the fingertip before the metabolically demanding exercise task, and then 1 min, 3 min, 5 min, 30 min and 60 min post-exercise. During the recovery after exercise, the participants were resting passively supine on a couch until the blood lactate analysis was finished (except for the conductance of the vertical jump test). After cleansing the fingertip with an alcohol swab and pricking it with a sterile lancet, 0.3 µL of blood was collected into a reagent strip of a pocket analyser (Pro2, Arkray Inc., Kyoto, Japan) for an immediate read-out of the lactate value.

Plasma malondialdehyde (MDA) concentration was analysed before incremental cycling and 1 h post-test. Blood samples were collected into EDTA-containing vacuum tubes by puncturing an antecubital vein. Plasma was then immediately harvested by 1500 g centrifugation for 15 min at 4 °C and stored at −80 °C. Spectrophotometric analysis (Spark 10M Tecan, Männedorf, Switzerland) was performed at 450 nm wavelength to measure MDA concentration using specialized kits (Ref. #E1371Hu, BT Lab, Jiaxing, China) according to the enclosed protocol description. Samples were analysed in duplicate, and average values were reported. The coefficient of variation of the assay was less than 10%.

### 2.6. Statistical Analysis

Descriptive statistics, presented as the mean ± standard deviation (SD), were calculated for each measured variable. The preliminary analysis of data distribution normality was conducted using the Shapiro–Wilk test. To assess the main and interaction effects of condition and time on blood lactate and countermovement jump (CMJ) height, a two-way repeated measures analysis of variance (RM ANOVA) was employed. When a statistically significant effect was identified, Tukey’s honestly significant difference (HSD) post hoc test was subsequently performed to conduct pairwise multiple comparisons and pinpoint specific mean differences. For any other comparisons involving paired measurements outside of the RM ANOVA structure, a dependent samples t-test was utilized to determine significant differences. Effect sizes were reported as partial eta squared (ηp^2^) for ANOVA analyses and Cohen’s *d* (paired-samples) for *t*-test results [37]. All statistical analyses were performed using IBM SPSS Statistics (version 30.0; IBM Corp., Armonk, NY, USA), with the threshold for statistical significance set a priori at *p* < 0.05.

## 3. Results

### 3.1. Baseline Measurements

No significant differences were observed between intervention periods in participants’ body composition or exercise performance (η^2^ < 0.05) (Table 1). The questionnaires revealed that only one participant used supplements (omega-3). Only a few participants consumed small amounts of broccoli-related foods (such as mustard or radish), while nearly one-third of the participants reported drinking 1–3 cups of coffee per day, and one participant reported drinking 2–3 cups of tea per day. The consumption of drinks was balanced between the intervention and placebo conditions.

### 3.2. 24-H Urine SFN Excretion

**Figure 2:**
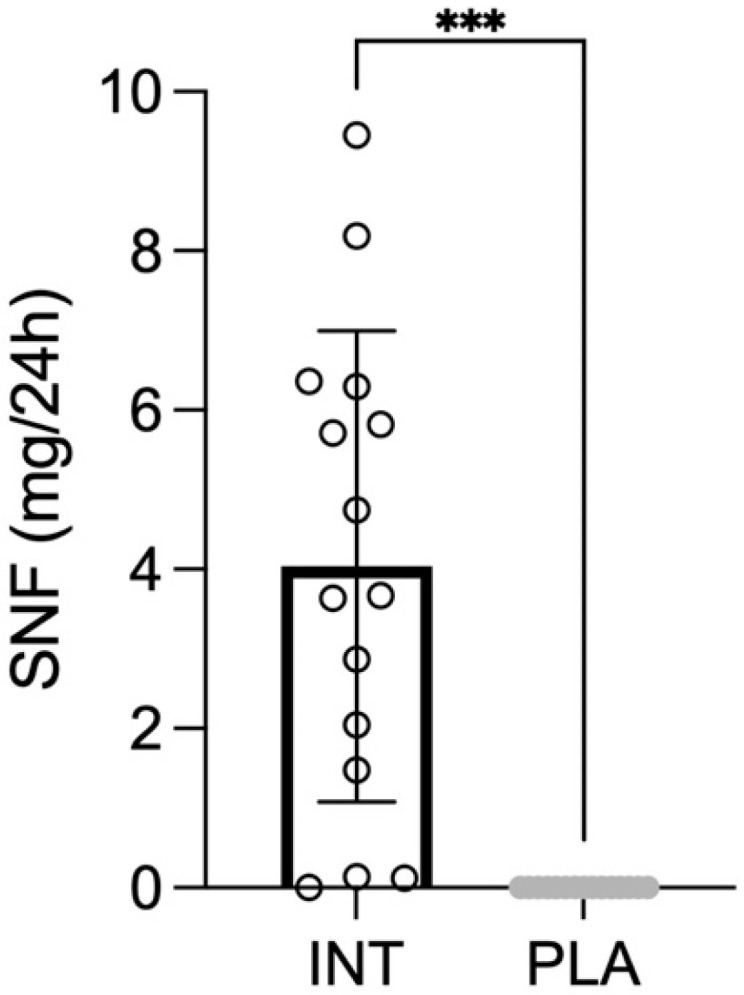
Total 24-h urinary sulforaphane (SFN) excretion in the intervention (INT) and placebo (PLA) arms. *** *p* < 0.001.

Urinary sulforaphane excretion was significantly higher following broccoli powder supplementation compared to placebo (*p* < 0.001, *d =* 1.93), confirming effective bioavailability of the compound (Figure 2).

### 3.3. MDA Concentration

**Figure 3:**
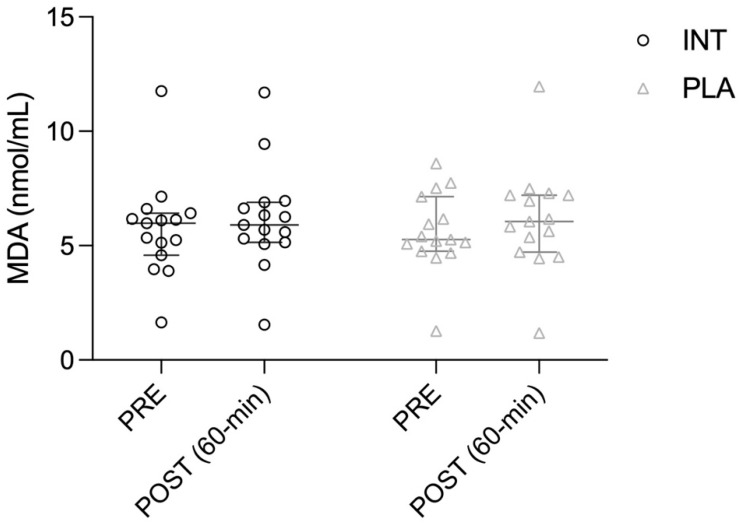
Plasma malondialdehyde (MDA) concentration (individual data points) measured pre- and post-exercise in the intervention (INT) and placebo (PLA) trials.

No significant effects were observed for Intervention (*p* = 0.949, η^2^ = 0.005), Time (*p* = 0.052, η^2^ = 0.156), or Time × Intervention interaction (*p* = 0.703, η^2^ = 0.001). MDA levels showed only a tendency to increase post-exercise, similarly in both trials (PLA: *p* = 0.099; INT: *p* = 0.154) (Figure 3).

### 3.4. Lactate Concentration Dynamics

**Figure 4:**
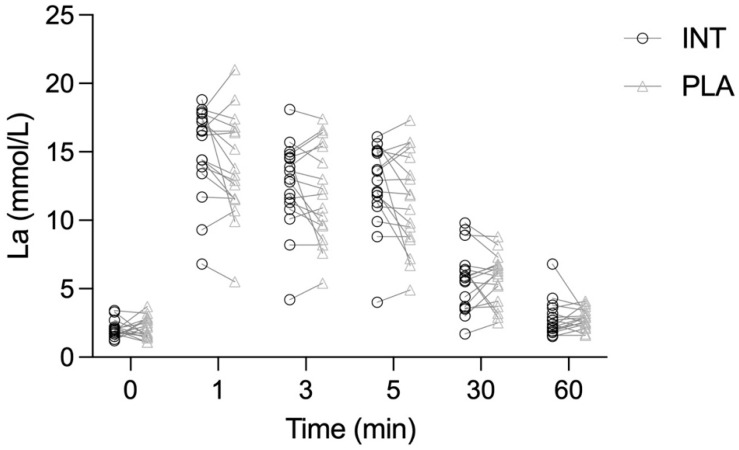
Blood lactate concentration (individual data points) measured from baseline to 60 min post-exercise in the intervention (INT) and placebo (PLA) trials.

Lactate concentration changed significantly over time in response to exercise (Time effect, *p* < 0.001, η^2^ = 0.904), but there was no significant Intervention effect (*p* = 0.501, η^2^ = 0.021) or Time × Intervention interaction (*p* = 0.544, η^2^ = 0.024), therefore mean lactate concentrations did not differ between the intervention and placebo conditions at any time point (Figure 4).

### 3.5. Muscle Power

**Figure 5:**
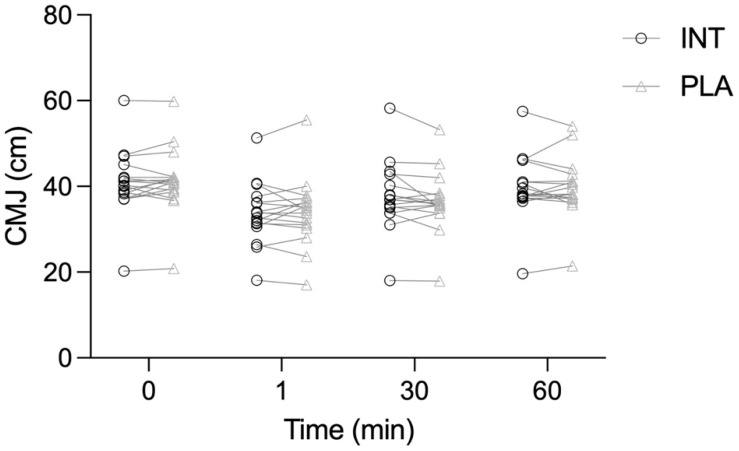
Countermovement jump (CMJ) height measured at baseline and post-exercise (1, 30, 60 min) in the intervention (INT) and placebo (PLA) trials.

CMJ height showed a significant main effect of Time (*p* < 0.001, η^2^ = 0.703), indicating transient decrements in performance following exercise, but neither the Intervention effect (*p* = 0.945, η^2^ = 0.002) nor the Time × Intervention interaction (*p* = 0.394, η^2^ = 0.030) was significant, with no differences between broccoli supplement and placebo detected at any time point (Figure 5).

## 4. Discussion

This study examined the effects of short-term broccoli powder supplementation, rich in SFN precursor glucoraphanin, on oxidative stress and performance responses to a single bout of intense aerobic exercise in healthy adults. The main findings were that despite SFN output in urine being markedly higher following broccoli powder supplementation, which confirms effective absorption and systemic availability of the compound, this did not influence exercise-induced changes in plasma MDA concentration, exercise test performance, or recovery measured by blood lactate and muscle power dynamics. Therefore, these results suggest that, under single-bout exercise conditions mimicking a short, intense aerobic training session, despite successful delivery of its bioactive component, broccoli powder supplementation does not acutely modify oxidative stress, metabolic response or recovery-related performance outcomes.

The absence of significant changes in plasma MDA likely reflects the relatively mild oxidative stress induced by the chosen exercise protocol [4,13]. However, a simulated 1500 m race induced similar blood lactate levels (similar metabolic stress) and a significant increase in plasma MDA in well-trained runners [38], suggesting that other exercise-related factors as the mode of activity, could play a role during this process. Of importance, the current study was designed to replicate realistic activity levels representative of non-athletic individuals, for whom the supplement is intended [21]. Within this ecologically valid model, endogenous antioxidant systems may have been sufficient to buffer exercise-induced ROS, leaving limited scope for further modulation by exogenous antioxidants [4,13,34]. Nonetheless, the urine SFN excretion data in response to the first dose indicate that concentrated broccoli preparation can effectively increase systemic exposure to this phytochemical in most individuals, supporting its potential for longer-term antioxidant or cytoprotective benefits under conditions of greater oxidative stress [24,25,34].

Significantly increased sulforaphane output through urine observed after broccoli powder supplementation in most (but not all) of the participants suggests the bioavailability and effective absorption of the compound for most cases, consistent with previous studies demonstrating rapid systemic appearance of sulforaphane following oral intake of glucoraphanin-rich preparations [24,25]. Non-responsiveness in terms of urinary sulforaphane excretion may, at least in part, reflect interindividual variability in gut microbiota composition [39], which plays a key role in the conversion of glucoraphanin to sulforaphane. This variability could therefore influence the bioavailability of sulforaphane from broccoli and other dietary sources. However, even after excluding participants whose urinary sulforaphane concentrations were below the detection limit, the overall study outcomes remained unchanged, with no differences observed between conditions in any of the measured parameters. Our findings, therefore, suggest that the lack of effect on oxidative stress marker MDA both before and after the exercise tasks in the present study is likely not because of the limited absorption of sulforaphane. Saying that, it is also fair to admit that urinary sulforaphane output could not well reflect the overall muscle expose with this compound. On the other hand, the metabolic and oxidative challenge elicited by the exercise was likely modest, allowing endogenous antioxidant systems to maintain redox homeostasis without requiring substantial additional buffering from exogenous compounds [3,4,13]. Under these circumstances, the ability of sulforaphane to further modulate lipid peroxidation (as indexed by MDA) or influence performance outcomes would understandably be limited.

It is important to recognize that sulforaphane exerts its antioxidant and cytoprotective effects primarily through activation of the Nrf2–ARE signaling pathway, a process that enhances the expression of endogenous antioxidant enzymes over time rather than providing immediate radical scavenging [24,26,34]. Therefore, the acute experimental design employed here may not fully capture the potential of sulforaphane supplementation, which might be more evident under conditions of repeated exercise sessions, prolonged supplementation, or higher oxidative stress loads. The absence of detectable differences in oxidative stress and performance parameters between the broccoli-supplemented *vs*. placebo conditions aligns with previous findings showing that metabolically demanding aerobic exercise induces only a transient and physiologically manageable increase in reactive oxygen species production [3,4,13]. In such conditions, the body’s intrinsic antioxidant mechanisms—including enzymatic defenses such as superoxide dismutase, catalase, and glutathione peroxidase—are typically sufficient to maintain redox balance. Therefore, additional antioxidant input through dietary supplementation may exert only marginal or undetectable effects.

From a methodological perspective, the choice to employ a realistic exercise protocol represents a deliberate and important distinction of the present study. While more strenuous or prolonged exercise bouts might have amplified oxidative stress responses and increased the likelihood of observing measurable effects of supplementation, this would have compromised the ecological validity of the research. Since broccoli and similar nutraceuticals are primarily marketed for general well-being and use by non-athletic individuals, the present protocol was purposefully designed to mirror everyday physical activity rather than experimental overload [22,25]. This approach allows for the evaluation of supplement efficacy under conditions that reflect typical consumer use, providing a more practical interpretation of its physiological relevance.

Taken together, the findings indicate that while broccoli powder preparation enriched with mustard seed powder effectively increases systemic sulforaphane bioavailability, its acute effects on oxidative stress modulation are unlikely to be evident in the context of a relatively mild exercise bout, where oxidative stress is mild and transient.

### Limitations and Future Perspectives

Several factors should be considered when interpreting the present findings. First, the design of the implemented study captures only the short-term responses to supplementation and only in relation to a single bout of exercise. Given the transcriptional nature of sulforaphane’s mechanism of action through Nrf2 activation, longer supplementation periods may be required to observe measurable upregulation of endogenous antioxidant enzymes and downstream effects [40]. Second, MDA, while widely used as a marker of lipid peroxidation, reflects only one aspect of oxidative stress; a broader panel of biomarkers—including reduced and oxidized glutathione ratio, antioxidant enzyme activities, and inflammatory markers—would provide a more comprehensive assessment of free radical stress [4,11,12]. In addition, MDA was measured at a single time point after exercise, which possibly did not allow for capturing differences in earlier post-exercise oxidative responses between the conditions.

Furthermore, the sample comprised healthy, recreationally active male participants with presumably well-functioning antioxidant systems, which may have limited the potential for further improvement through supplementation. Future studies could explore populations with higher baseline oxidative stress levels, such as sedentary, older, metabolically challenged, or hazardous environment-exposed individuals, in whom sulforaphane potential might be disclosed more evidently [17,35]. Finally, assessing chronic supplementation during regular physical training could help determine whether sustained Nrf2 activation translates into improved red-ox homeostasis, mitigated exercise-induced damage, reduced frequency and severity (duration) of infections, or enhanced recovery capacity [34].

## 5. Conclusions

In conclusion, the present study demonstrates that dietary supplementation of broccoli powder enriched with myrosinase-containing mustard seed powder effectively enhances urinary sulforaphane metabolite excretion, indicating successful systemic absorption. However, short-term consumption of the supplement does not seem to acutely influence oxidative and metabolic stress, performance and recovery after an acute bout of metabolically challenging aerobic exercise in untrained healthy men. Disclosure of the antioxidant potential of sulforaphane may require a larger oxidative challenge to elicit measurable modulatory effects of the broccoli supplementation.

## Figures and Tables

**Table 1:** Characteristics of the study population with differences in terms of body composition and exercise protocol parameters between the two interventions (INT: intervention; PLA: placebo). Data are presented as mean ± SD.

	INT (*n* = 17)	PLA (*n* = 17)	*p*
Age (y)	23 ± 5		
BM (kg)	80 ± 13	80 ± 12	0.40
H (cm)	182 ± 6		
BF (%)	15 ± 5	15 ± 4	0.97
VO_2max_ (L/min)	3.7 ± 0.6	3.6 ± 0.6	0.43
VO_2max_ (mL/min/kg)	47 ± 10	46 ± 9	0.56
HR_max_ (bpm)	184 ± 10	182 ± 12	0.38
PPO (W)	280 ± 45	276 ± 45	0.23

Notes: BM: body mass, H: height, BF: body fat, VO_2max_: maximal oxygen uptake, HR_max_: peak heart rate, PPO: peak power output.

## Data Availability

The original contributions presented in this study are included in the article. Further inquiries can be directed to the corresponding authors.

## Abbreviations

The following abbreviations are used in this manuscript:

**Abbreviation**	**Definition**
ANOVA	Analysis of variance
ARE	Antioxidant response element
BCAA	Branched-chain amino acids
BF	Body fat
BM	Body mass
CC BY	Creative Commons Attribution
CMJ	Countermovement jump
CO_2_	Carbon dioxide
EDTA	Ethylenediaminetetraacetic acid
HR	Heart rate
HRmax	Maximal heart rate
HSD	Honestly significant difference
INT	Intervention
LC-MS	Liquid chromatography–mass spectrometry
MDA	Malondialdehyde
NQO1	NAD(P)H quinone oxidoreductase 1
Nrf2	Nuclear factor erythroid 2–related factor 2
PLA	Placebo
PPO	Peak power output
RER	Respiratory exchange ratio
RM ANOVA	Repeated-measures analysis of variance
ROS	Reactive oxygen species
SD	Standard deviation
SFN	Sulforaphane
VE	Pulmonary ventilation
VO_2_	Oxygen consumption
VO_2_max	Maximal oxygen uptake

## References

[1] Powers S.K., Jackson M.J. (2008). Exercise-Induced Oxidative Stress: Cellular Mechanisms and Impact on Muscle Force Production. Physiol. Rev..

[2] Place N., Ivarsson N., Venckunas T., Neyroud D., Brazaitis M., Cheng A.J., Ochala J., Kamandulis S., Girard S., Volungevičius G. (2015). Ryanodine Receptor Fragmentation and Sarcoplasmic Reticulum Ca2+ Leak after One Session of High-Intensity Interval Exercise. Proc. Natl. Acad. Sci. USA.

[3] Powers S.K., Deminice R., Ozdemir M., Yoshihara T., Bomkamp M.P., Hyatt H. (2020). Exercise-Induced Oxidative Stress: Friend or Foe?. J. Sport Health Sci..

[4] Thirupathi A., Wang M., Lin J.K., Fekete G., István B., Baker J.S., Gu Y. (2021). Effect of Different Exercise Modalities on Oxidative Stress: A Systematic Review. Biomed Res. Int..

[5] Jomova K., Raptova R., Alomar S.Y., Alwasel S.H., Nepovimova E., Kuca K., Valko M. (2023). Reactive Oxygen Species, Toxicity, Oxidative Stress, and Antioxidants: Chronic Diseases and Aging. Arch. Toxicol..

[6] Chandimali N., Bak S.G., Park E.H., Lim H.-J., Won Y.-S., Kim E.-K., Park S.-I., Lee S.J. (2025). Free Radicals and Their Impact on Health and Antioxidant Defenses: A Review. Cell Death Discov..

[7] Jena A.B., Samal R.R., Bhol N.K., Duttaroy A.K. (2023). Cellular Red-Ox System in Health and Disease: The Latest Update. Biomed. Pharmacother..

[8] Cheng A.J., Yamada T., Rassier D.E., Andersson D.C., Westerblad H., Lanner J.T. (2016). Reactive Oxygen/Nitrogen Species and Contractile Function in Skeletal Muscle during Fatigue and Recovery. J. Physiol..

[9] Allen D.G., Lamb G.D., Westerblad H. (2008). Skeletal Muscle Fatigue: Cellular Mechanisms. Physiol. Rev..

[10] Richards A.J., Watanabe D., Yamada T., Westerblad H., Cheng A.J. (2025). Task-Dependent Mechanisms Underlying Prolonged Low-Frequency Force Depression. Exerc. Sport Sci. Rev..

[11] Mason S.A., Trewin A.J., Parker L., Wadley G.D. (2020). Antioxidant Supplements and Endurance Exercise: Current Evidence and Mechanistic Insights. Redox Biol..

[12] Clemente-Suárez V.J., Bustamante-Sanchez Á., Mielgo-Ayuso J., Martínez-Guardado I., Martín-Rodríguez A., Tornero-Aguilera J.F. (2023). Antioxidants and Sports Performance. Nutrients.

[13] Taherkhani S., Valaei K., Arazi H., Suzuki K. (2021). An Overview of Physical Exercise and Antioxidant Supplementation Influences on Skeletal Muscle Oxidative Stress. Antioxidants.

[14] Wang Y., He Z., Long C., Li Y., Yuan Y., Huang T. (2025). Systematic Review and Meta-Analysis of Antioxidants with or without Exercise Training Improving Muscle Condition in Older Adults. Sci. Rep..

[15] Gomez-Cabrera M.C., Ristow M., Viña J. (2012). Antioxidant Supplements in Exercise: Worse than Useless?. Am. J. Physiol.-Endocrinol. Metab..

[16] Bruns D.R., Ehrlicher S.E., Khademi S., Biela L.M., Peelor F.F., Miller B.F., Hamilton K.L. (2018). Differential Effects of Vitamin C or Protandim on Skeletal Muscle Adaptation to Exercise. J. Appl. Physiol..

[17] Tkaczenko H., Kurhaluk N. (2025). Antioxidant-Rich Functional Foods and Exercise: Unlocking Metabolic Health Through Nrf2 and Related Pathways. Int. J. Mol. Sci..

[18] Babbar R., Dhiman A., Sethi K. (2025). Natural Bioactive Compounds in Cardiovascular Protection: Flavonoids, Alkaloids, and Carotenoids in Focus. ChemistrySelect.

[19] Bouyahya A., Bakrim S., Aboulaghras S., El Kadri K., Aanniz T., Khalid A., Abdalla A.N., Abdallah A.A., Ardianto C., Ming L.C. (2024). Bioactive Compounds from Nature: Antioxidants Targeting Cellular Transformation in Response to Epigenetic Perturbations Induced by Oxidative Stress. Biomed. Pharmacother..

[20] Flockhart M., Nilsson L.C., Tillqvist E.N., Vinge F., Millbert F., Lännerström J., Nilsson P.H., Samyn D., Apró W., Sundqvist M.L. (2023). Glucosinolate-Rich Broccoli Sprouts Protect against Oxidative Stress and Improve Adaptations to Intense Exercise Training. Redox Biol..

[21] Andrés C.M.C., Pérez de la Lastra J.M., Munguira E.B., Juan C.A., Pérez-Lebeña E. (2025). The Multifaceted Health Benefits of Broccoli—A Review of Glucosinolates, Phenolics and Antimicrobial Peptides. Molecules.

[22] Palliyaguru D.L., Yuan J.-M., Kensler T.W., Fahey J.W. (2018). Isothiocyanates: Translating the Power of Plants to People. Mol. Nutr. Food Res..

[23] Vanduchova A., Anzenbacher P., Anzenbacherova E. (2019). Isothiocyanate from Broccoli, Sulforaphane, and Its Properties. J. Med. Food.

[24] Dmytriv T.R., Lushchak O., Lushchak V.I. (2025). Glucoraphanin Conversion into Sulforaphane and Related Compounds by Gut Microbiota. Front. Physiol..

[25] Baldelli S., Lombardo M., D’Amato A., Karav S., Tripodi G., Aiello G. (2025). Glucosinolates in Human Health: Metabolic Pathways, Bioavailability, and Potential in Chronic Disease Prevention. Foods.

[26] Merchant H.J., Forteath C., Gallagher J.R., Dinkova-Kostova A.T., Ashford M.L.J., McCrimmon R.J., McNeilly A.D. (2025). Activation of the Nrf2 Pathway by Sulforaphane Improves Hypoglycaemia-Induced Cognitive Impairment in a Rodent Model of Type 1 Diabetes. Antioxidants.

[27] Loboda A., Damulewicz M., Pyza E., Jozkowicz A., Dulak J. (2016). Role of Nrf2/HO-1 System in Development, Oxidative Stress Response and Diseases: An Evolutionarily Conserved Mechanism. Cell Mol. Life Sci..

[28] Li L., Dong H., Song E., Xu X., Liu L., Song Y. (2014). Nrf2/ARE Pathway Activation, HO-1 and NQO1 Induction by Polychlorinated Biphenyl Quinone Is Associated with Reactive Oxygen Species and PI3K/AKT Signaling. Chem.-Biol. Interact..

[29] Holman J., Hurd M., Moses P.L., Mawe G.M., Zhang T., Ishaq S.L., Li Y. (2023). Interplay of Broccoli/Broccoli Sprout Bioactives with Gut Microbiota in Reducing Inflammation in Inflammatory Bowel Diseases. J. Nutr. Biochem..

[30] Dwibedi C., Axelsson A.S., Abrahamsson B., Fahey J.W., Asplund O., Hansson O., Ahlqvist E., Tremaroli V., Bäckhed F., Rosengren A.H. (2025). Effect of Broccoli Sprout Extract and Baseline Gut Microbiota on Fasting Blood Glucose in Prediabetes: A Randomized, Placebo-Controlled Trial. Nat. Microbiol..

[31] Kaczmarek J.L., Liu X., Charron C.S., Novotny J.A., Jeffery E.H., Seifried H.E., Ross S.A., Miller M.J., Swanson K.S., Holscher H.D. (2019). Broccoli Consumption Affects the Human Gastrointestinal Microbiota. J. Nutr. Biochem..

[32] Scheiman J., Luber J.M., Chavkin T.A., MacDonald T., Tung A., Pham L.-D., Wibowo M.C., Wurth R.C., Punthambaker S., Tierney B.T. (2019). Meta-Omics Analysis of Elite Athletes Identifies a Performance-Enhancing Microbe That Functions via Lactate Metabolism. Nat. Med..

[33] Komine S., Miura I., Miyashita N., Oh S., Tokinoya K., Shoda J., Ohmori H. (2021). Effect of a Sulforaphane Supplement on Muscle Soreness and Damage Induced by Eccentric Exercise in Young Adults: A Pilot Study. Physiol. Rep..

[34] Ruhee R.T., Ma S., Suzuki K. (2025). Effects of Sulforaphane Treatment on Skeletal Muscle from Exhaustive Exercise-Induced Inflammation and Oxidative Stress Through the Nrf2/HO-1 Signaling Pathway. Antioxidants.

[35] Saeidi A., Soltani M., Daraei A., Nohbaradar H., Haghighi M.M., Khosravi N., Johnson K.E., Laher I., Hackney A.C., VanDusseldorp T.A. (2021). The Effects of Aerobic-Resistance Training and Broccoli Supplementation on Plasma Dectin-1 and Insulin Resistance in Males with Type 2 Diabetes. Nutrients.

[36] Jamurtas A.Z. (2018). Exercise-Induced Muscle Damage and Oxidative Stress. Antioxidants.

[37] Ferguson C.J. (2009). An Effect Size Primer: A Guide for Clinicians and Researchers. Prof. Psychol. Res. Pract..

[38] Venckunas T., Minderis P., Silinskas V., Buliuolis A., Maughan R.J., Kamandulis S. (2024). Effect of Low vs. High Carbohydrate Intake after Glycogen-Depleting Workout on Subsequent 1500 m Run Performance in High-Level Runners. Nutrients.

[39] Bouranis J.A., Beaver L.M., Wong C.P., Choi J., Hamer S., Davis E.W., Brown K.S., Jiang D., Sharpton T.J., Stevens J.F. (2024). Sulforaphane and Sulforaphane-Nitrile Metabolism in Humans Following Broccoli Sprout Consumption: Inter-Individual Variation, Association with Gut Microbiome Composition, and Differential Bioactivity. Mol. Nutr. Food Res..

[40] Houghton C.A., Fassett R.G., Coombes J.S. (2016). Sulforaphane and Other Nutrigenomic Nrf2 Activators: Can the Clinician’s Expectation Be Matched by the Reality?. Oxidative Med. Cell. Longev..

